# Assessing the feasibility of the GOTT (Gabapentinoid and Opioid Tapering Toolkit) in a primary care setting in North-East England

**DOI:** 10.1177/20494637241291534

**Published:** 2024-10-20

**Authors:** Lucy Johnson, Frances Cole, Rebecca Kinchin, Andrea Francis, Konrad Winiarek, Kate Hampshire, Paul Chazot

**Affiliations:** 1 Department of Anthropology, 3057 Durham University, Durham, UK; 2 Live well with Pain; Wolfson Research Institute for Health and Wellbeing, 3057 Durham University, Durham, UK; 3 Clifton Court GP Practice, Darlington Promary Care Network, England, UK; 4 Department of Biosciences, Wolfson Research Institute for Health and Wellbeing, 3057 Durham University, Durham, UK

**Keywords:** Biopsychosocial, chronic pain, confidence, pain management, primary care, self-management, LWWP 10-Footsteps

## Abstract

**Objective:**

To assess the feasibility and possible impacts of implementation of systematic non-pharmacological interventions to reduce the level of prescribing of opioid and gabapentinoid analgesics for chronic non-cancer pain (CNCP), particularly high-dose prescriptions, through a proof-of-concept study in a deprived area (second lowest decile) primary care practice in North-East England.

**Participant:**

Twenty-five primary care staff (clinical and non-clinical) of which 18 clinicians received the intervention.

**Intervention used in this study practice known as GOTT (Gabapentinoid and Opioid Toolkit):**

All clinicians received an educational skills programme to support patient pain self-management, tailored on the clinicians’ self-assessment of their learning needs, embedding both clinician skill learning and patient self-care resources for rapid access within consultations into a GP clinical management computer system.

**Outcome measures:**

Clinical staff completed questionnaires before and after the GOTT intervention to assess levels of knowledge and confidence in their own skills to support chronic pain self-management across several domains. Prescription data were used to measure changes in opioid and gabapentinoid prescribing at the practice across the 12-month intervention and 30-month follow-up period.

**Results:**

Prescribing of opioid and gabapentinoid/pregabalin decreased substantially in the practice across the intervention period (c. 90% in high-dose opioid [*p* = .0118], and 15% gabapentin/pregabalin prescriptions, respectively), over a one-year period during the COVID-19 pandemic. Follow-up analysis showed 100% and c.50% reductions, respectively, in December 2022. The questionnaire data showed an increase in clinician confidence in skills to enable self-management over the intervention period, overall (*p* = .044) and, specifically across three of the five domains measured: supporting behavioural change (*p* = .028), supporting self-care (*p* = .008), and managing difficult consultations (*p* = .011).

**Conclusion:**

The GOTT intervention program provided some initial evidence of a proof-of-concept for the implementation of a systematic non-pharmacological pain management skills and resources programme addressing lack of confidence in skills to introduce and support self-management and reduce use of strong opioids and gabapentinoids.

## Introduction

Chronic pain affects approximately 10% of the world’s population, and its management presents a major global health challenge to clinicians and patients alike.^1^ Growing concerns around the provision of effective chronic pain management, particularly in higher-income countries, have collided with concerns around increased access to strong (opioid and gabapentinoid) painkillers and associated harms, including risk of dependence, addiction, and death through overdose.^2^ The gabapentinoids, gabapentin and pregabalin, are primarily anticonvulsants although both have been approved, until recently, for use in neuropathic pain and are widely used off-label for general chronic pain management.^3,4^

Opioids and gabapentinoids are now not recommended for the treatment of chronic primary pain, which is better managed by a specialist Multi-Disciplinary Team (MDT) using a person-centred approach to treatment.^5,6^ Recent guidelines introduced by the National Institute for Health and Care Excellence (NICE) in 2021 state that *no* painkillers should be routinely prescribed for the management of chronic non-cancer pain (CNCP) and advocate for the non-pharmacological management of chronic primary pain conditions in all primary care settings (NICE, 2021). In England, prior to the GOTT programme, there are stark regional and socio-economic differences in opioid painkiller prescriptions,^5^ with rurality and poverty associated with increased prevalence of both chronic pain and opioid use.^7,8^ There is thus a pressing need, particularly in areas of high opioid use, to develop resources that can be used in primary care settings to provide more confidence in delivering more effective person-centred care for those living with chronic pain.^9,10^

**Figure 1.**
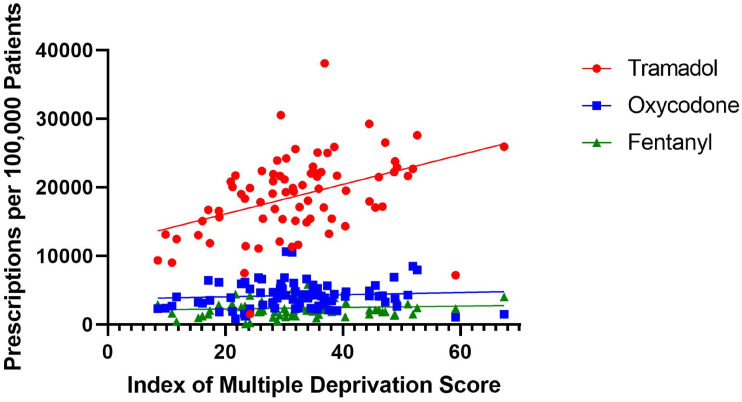
Highly significant correlation between Tramadol prescriptions and multiple deprivation score (R^2^ = 0.1767, *p* = .0001).

The GOTT (Gabapentinoid and Opioid Tapering Toolkit) was developed in response to the need to provide a person-centred, biopsychosocial approach to chronic pain management in primary care.^9^ The toolkit aims were to enable clinicians to support patients with chronic pain using a range of pain self-management knowledge and skills, and safer or reduced prescribing of opioid and gabapentinoid analgesics in a sustainable and cost-effective way. It was developed and implemented at a GP practice in a deprived area in Northeast England (Figure 1).

The NE has among the highest prevalence both of chronic pain and opioid/gabapentinoid prescriptions in the country (Mordecai *et al.*, 2018; Todd *et al.*, 2018), and this practice studied was at the upper end, with one of highest prescription rates of opioids in County Durham.

This study took place between June 2020 and June 2021, with further follow-up assessment in December 2022. It involved the systematic development and distribution of novel online pain management learning materials (Live Well with Pain, 2023) in a large challenging GP practice (list size 11,850) in the North East. These materials were made accessible to clinical and non-clinical staff, but training was primarily aimed towards those usually involved in pain management consultations, especially GPs. The success of the intervention was then assessed using a mixed-methods approach, in order to gain a holistic overview of what worked (and what did not) during this feasibility proof-of-concept study.

## Research questions

The proof-of-concept study set out to address the following research questions:

1. What were the clinicians’ current levels of confidence in their own knowledge, skills, and use of tools and resources to support self-management within a primary care setting as part of a opioid deprescribing approach in a high opioid and gabapentinoid prescribing practice?
2. What were the key factors in the clinician approaches to pain management; both self-management and high prescribing of medication of opioids and gabapentinoids prior to and after the intervention using qualitative approaches?
3. What was the outcome of the GOTT intervention process, following a tailored educational programme based on their learning needs, on the clinician confidence levels to engage patients in self-management, and on face-to-face or via telephone conversations, to address deprescribing of opioids and gabapentinoids? The intervention also included the integration of self-management resources and a structural consultation process within the clinical System One GP computer system.

## Methods

### Study design

A mixed-methods approach was employed to assess the potential quantitative and qualitative impact of the GOTT intervention on primary care clinicians, nurses, and pharmacists, evaluating their confidence in knowledge and skills in supporting self-management and change in *prescribing practices and opioid/gabapentinoid prescription rates in the practice.*

Anonymised data covering a time period from August 2018, through to August 2020 to December 2022, was collected from OpenPrescribing.net (OpenPrescribing.net, EBM DataLab, University of Oxford, 2020). These data included the numbers of prescriptions of each drug, corrected for practice size, as well as the doses at which these drugs were prescribed. Several different parameters were then used to assess the efficacy of the GOTT programme in reducing prescriptions. Firstly, the prescription rates of gabapentinoids and opioids were tracked through time. Data was collected for both clinically approved gabapentinoids, gabapentin and pregabalin, as well as three opioids, tramadol, oxycodone, and fentanyl. These opioids were chosen as they belong to three different opioid strength classes: low, medium, and high, respectively. This allows the opioids studied to approximately represent all opioids prescribed at the test GP practice. Secondly, importantly, the proportions of all opioid prescriptions classed as high dose were tracked. OpenPrescribing.net uses opioid strength conversion calculations to determine the proportion of all opioids prescribed at doses above 120 mg morphine equivalents (MMEs). Finally, the number of gabapentinoids prescribed at each dose was collected. This number was then converted to the percentage of all prescriptions of that medication. (b) The learning needs assessment questionnaire was undertaken with all clinical groups before the intervention and 9 months after educational programme and clinical assessment process intervention changes. (c) qualitative assessment was undertaken in short semi-structured interviews with 18 non-clinicians and clinicians within the practice.

### Ethics

Ethical approval for this study was granted by [Department of Anthropology, Durham University] (reference: ANTH-2021-06-03T11_57_48-kggd78). Information sheets were given to practitioners via email and physically in the practice. Verbal consent was taken prior to interviews undertaken by co-author LJ, and consent to record interviews was taken prior to recording and again at the end of the interview. Interviews were designed to be quick and unobtrusive, so to avoid becoming a time burden on participants. Participant participation was voluntary, and they could withdraw consent to participate at any point. Procedures for ensuring anonymity and confidentiality were made explicit prior to interviews.

### Details of the intervention components

• Clinician and non-clinician training programme: group based and self-directed learning.
• Rapid access in and outside consultations in use of self-management and medicine management information resources and decision aid tools.

• Engagement of non-clinical practice staff in support of person-centred pain management and service access.

It involved a systematic assessment of learning needs of clinicians to inform the content developed for the educational programme. This programme was a mixture of face-to-face and online pain management learning sessions and materials as identified by clinicians to address own knowledge and skills gap within the primary care. This programme was largely designed by Dr Frances Cole external to the practice, the study co-author who had extensive training and service development experience in primary and community service care over 35 years, and co-authorship of self-help resources/books for people with pain and practitioners.

#### Training content consisted of two approaches

Eight hours: four two-hour online sessions on pain self-management training for clinicians. It included core skills of health coaching communication with behavioural activation skills of pacing, goal setting and conversations in consultations, on managing a chronic pain condition, and its impact on health and managing setbacks. These sessions were delivered by primary care clinicians skilled in health coaching training and pain management. Each session was recorded in audiovisual format for subsequent participant access.

GOTT Ten Footsteps practitioner programme https://livewellwithpain.co.uk/practitioner-resources/10-footsteps/ was developed to address learning needs identified from the self-assessment questionnaire (Table 1). The programme supported self-directed learning over a 12-week period. Every 2 weeks, the clinicians were directed by the study team, via email, to explore the key pain management skills linked to resources in learning areas located in the primary care practice computer system, System One: pacing, goal setting, increasing physical activity, sleep well, use of decision aid tools for medicine management and set-back management, acceptance, enabling people to understand the ‘the importance of brain pathways in pain and its management’, anxiety and depression moods related to pain. These all took place in the COVID-19 pandemic first wave June 2020.

**Table 1.** Confidence and knowledge items in the questionnaire.

Area	Domains	Items
Knowledge of	Chronic pain in relation to ICD-11 criteria *6 items, Likert scale*	
		Severe pain is always due to severe tissue damage
		Chronic pain is a disease in its own right
		If pain lasts for more than 3 months, it is better to avoid movements if they cause pain
		A person should not return to work until they are pain free
		Myofascial (muscle pain) commonly causes pain to be felt in other parts of the body in addition to its source
		Chronic pain is pain that lasts for more than 1 month
	The safe use of pain medication *10 items, Likert scale*	The WHO analgesic ladder was developed for patients with musculoskeletal pain
		Pregabalin improves pain and function in acute sciatica
		Opioids are highly effective at reducing pain in chronic non-cancer pain
		Opioids are effective at improving function and reducing disability in non-cancer pain
		Prescribing an opioid with pregabalin is appropriate
		Gabapentin and opioids do not interact pharmacologically
		Prescribing an opioid with tricyclic antidepressants is safe
		Prescribing a benzodiazepine with an opioid can be justified
		Gabapentin can be addictive
		Switching to a gabapentinoid from opioids is appropriate
	Supporting patient self-care entails *10 items, Likert scale*	Understanding the patient’s pain beliefs
		Telling the patient what to do to manage their pain*
		Helping patients to overcome setbacks they may find
		Supporting the patient to identify what they can do themselves
		Supporting the patient in managing their relationships*
Confidence in	Supporting patients in behaviour change *4 items, Likert scale*	Using pacing skills to manage daily activities
		Setting goals and giving self-rewards
		Becoming more physically active/fitter
		Healthy eating and lifestyle changes
	Supporting patients in managing mental health and mood issues *4 items, Likert scale*	Anxiety and stress
		Depression or low mood
		Anger moods
		Using pacing skills to manage daily activities
	Supporting patients in self-care *3 items, Likert scale*	Setting goals and giving self-rewards
		Becoming more physically active/fitter
		Healthy eating and lifestyle changes
	Dealing with difficult consultations *2 items, Likert scale*	Working with angry patients
		Working with demotivated patients
	Confidence as a practice team *2 items, Likert scale*	In using agreed practice guidelines and resources to enable patients manage their pain condition
		In implementing and adhering to a pain management plan

#### Access to pain management resources: Self-management and medicine decision aid tools in collaboration with practice lead GP and IT support personnel

a. The support of patient use of self-management skills **within** the consultation was addressed by improving access for the clinician to chronic pain-related self-management tools and resources, specific for patient use. These resources became the Patient Live Well with Pain Ten Footsteps programme and were embedded within the primary care computer system for rapid clinical access and clinical record use.
b. System One computer pain management template was developed with Read Coding to enable audit and health outcome tracking, and was implemented in collaboration with the Practice IT management and administrative staff.

This template reflected:

• the health function-focused Live Well With Pain (LWWP) Health Check template of four steps, a self-complete resource prior to their pain management assessment or review.
• Medicine review and agreed decision process with change in medication use.

The patient self-complete questionnaire known as The Live Well with Pain Health Check tool was linked to the project created System One template and recorded patient health need priorities, based on the impact of pain on patient’s perceived health and well-being exploring the steps that included

• 13 functional areas of health function based on SF-36 Health Status measure, physical, emotional, and social with health need area priorities,
• pain self-efficacy via the PSEQ2, and^11^
• mental well-being with WHO-5 (WHO, 1998) and pain distress and intensity using a Visual Analogue Scale from 0 to 10.^12^

This LWWP Health Check System One template in the patient computer clinical record reflected the key health impact areas of pain and principles of care in NICE Guidance 173 (Supplemental Figure 2). This questionnaire was part of the individual’s preparation for their Live Well with Pain and Medicines Management review with the clinician (Supplemental Figure 2).

3. Engagement through face-to-face sessions with non-clinical staff to support their knowledge of role of pain management and support patient access within the practice services.

### Data collection procedures

Self-assessment of knowledge and skills questionnaires were administered to all clinicians before and 12 months after the described intervention, to assess knowledge and confidence gained in relation to different areas of pain management, self-care, and medicine management.

Participants were asked to complete 21 Likert-scale questions to assess knowledge, grouped into three domains: chronic pain in relation to International Classification of Diseases (ICD-11) criteria (6 items), safe use of pain medication (10 items), and supporting patient self-care (5 items).

Participants were asked to assess their confidence in skills supporting patients across 5 domains: supporting behaviour change (4 items), managing mental health/mood (4 items), supporting self-care (3 items), managing difficult consultations (2 items), and confidence as a practice team (2 items): see Table 1. Altogether, 18 clinicians filled in at least one questionnaire at some point across the study.

Anonymised prescription data for the GP practice were sourced for the duration of the study period via openprescribing.net (OpenPrescribing.net, EBM DataLab, University of Oxford, 2020), to enable changes to be tracked through time. Data were collected for both gabapentinoids (gabapentin and pregabalin) and three opioids (tramadol, oxycodone, and fentanyl). These opioids were chosen as they belong to three different opioid strength classes (low, medium, and high respectively), thus representing the full range of opioid strengths prescribed at the GP practice. Secondly, the proportions of all opioid prescriptions classed as ‘high-dose’ were tracked. A high-dose opioid (HDO) prescription is one equivalent to more than 120 MME. This value was calculated by OpenPrescribing.net. Doses above this threshold are associated with a greatly increased risk of severe side effects without any measurable benefit.^13^ Doses of gabapentinoids prescribed were also tracked over time (unlike HDOs, this figure was not generated automatically, so the data were gathered and analysed manually).

Qualitative semi-structured interviews were conducted with both clinicians before and after the intervention period. A total of 25 staff members who held various clinical and non-clinical roles across the practice were interviewed and six clinical staff members were interviewed both before and after the intervention. Interviews were carried out either on Zoom or face-to-face (in June 2021) by the LJ author and audio recorded using a laptop and stored in an encrypted file. The interview guide covered these themes: experiences of previous chronic pain consultations and participants’ confidence around using non-pharmacological methods to help patients to manage chronic pain. Interviews with non-clinical (administrative/reception) staff were carried out post-intervention only, as the importance of the role of such staff to the intervention engagement only became apparent as the research progressed.

### Analysis

Questionnaire data (knowledge and confidence) were analysed using SPSS 28.0. Following descriptive statistics on each outcome variable, aggregate scores for each domain (i.e. knowledge regarding chronic pain and use of medicines) were produced by taking the mean of all items in that domain, for 2020 and 2021, respectively. Non-parametric statistical tests (Wilcoxon’s signed rank tests) were then employed to assess changes in each domain of knowledge and confidence in treating chronic pain before and after the intervention, with *p*-values of below 0.05 (2-sided) considered significant.

Analysis of prescription data (changes over time) was performed using GraphPad Prism version 9.0.0. For the regression analysis, dates were converted into number of weeks since the first data point. The gradients and intercepts of the lines formed by regression analysis were compared using an Analysis of Covariance (ANCOVA) test to determine if the differences were significant. For regression analysis, *p*-values of <0.05 were considered statistically significant, and all errors are reported as ± 1 SE unless otherwise stated.

Semi-structured interviews were transcribed and then thematically analysed using Scrivener software (v.3.1.5). Transcripts were coded and recoded by LJ using a grounded theory approach (Strauss and Corbin, 1990), generating four core themes: practical considerations, managing patient consultations, knowledge gaps, and impact of the COVID-19 pandemic.

### Patient involvement

The original intention was to convene a consultative group of patients for the direction of the study period. However, in the context of the COVID-19 pandemic, this was not possible. Three lived experiences of pain individuals were consulted on the GOTT 10-Footsteps and training online materials content during the development period.

## Results

### Questionnaire data

Data on clinicians’ knowledge and confidence in management of chronic pain are shown in Table 2. A total of 20 practitioners were asked to participate in the study; 12 prescribers comprising 9 GPs, 1 nurse practitioner prescriber, 1 practice nurse prescriber, and 1 clinical practitioner. Non-prescribers included four practice nurses, one pharmacist, one pharmacist technician, and three healthcare assistants. Response to the questionnaire was between 10 and 15 practitioners depending on the question.

**Table 2.** Changes in clinicians’ knowledge and confidence scores, June 2020 versus June 2021.

Area	Domains	June 2020 Likert scores	June 2021 Likert scores	Wilcoxon paired test *p*-values (2-sided)
Knowledge	Chronic pain (ICD-11 criteria)	3.90	4.17	0.056
	Safe use of pain medication	3.71	3.58	0.918
	Supporting patient self-care	3.84	4.19	0.032*
	*All knowledge*	3.86	3.81	0.260
Confidence	Supporting patients in behaviour change	3.09	3.91	0.028*
	Supporting patients in managing mental health and mood	3.34	3.32	0.161
	Supporting patients in self-care	2.11	2.87	0.008**
	Dealing with difficult consultations	2.64	3.36	0.011*
	Confidence as a practice team	2.54	3.21	0.064
	*All confidence*	2.87	3.60	0.044*

Significance (two-tailed): *p < .05; **p < .01.

**Figure 2.**
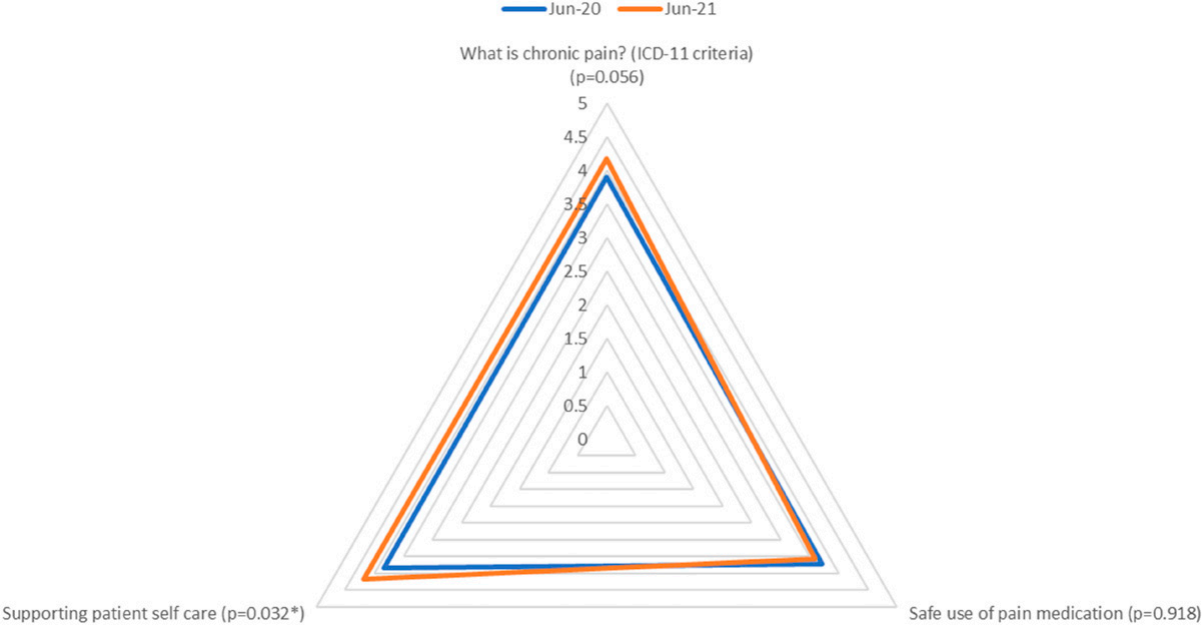
Spider graph demonstrating the changes in different domains of participant knowledge to provide different aspects of pain management in primary care.

**Figure 3.**
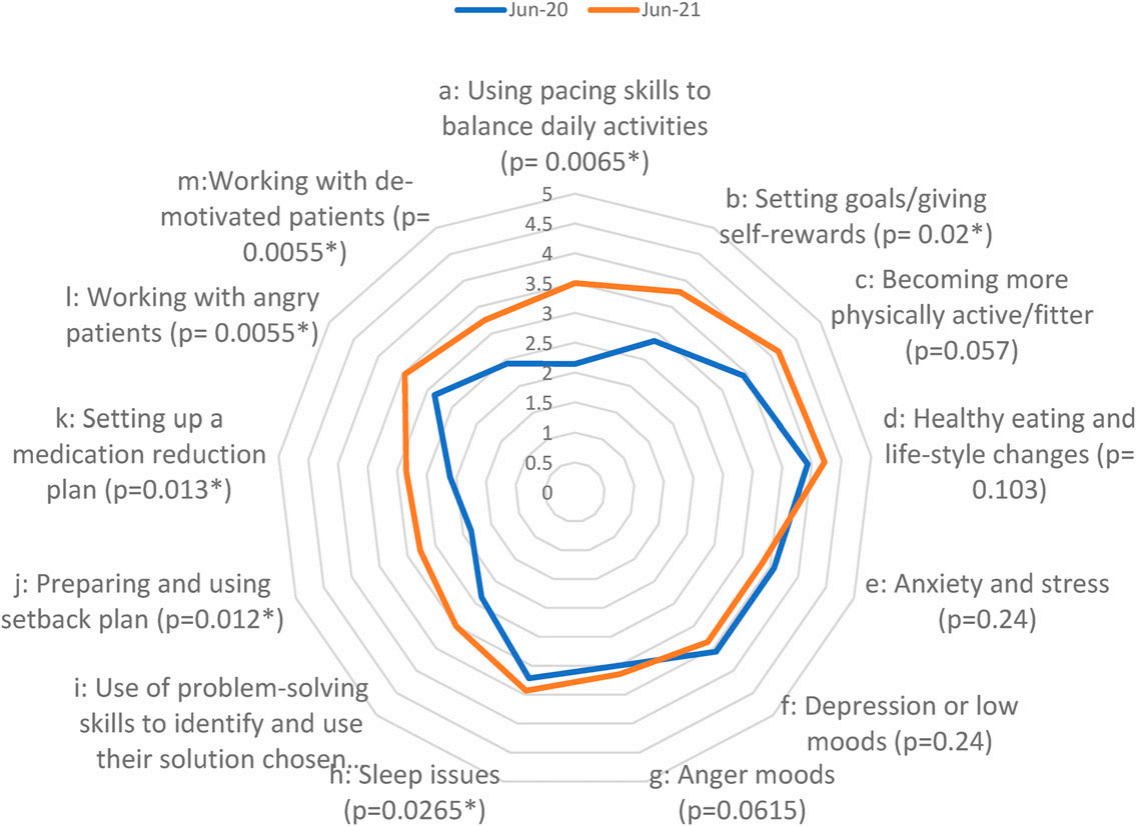
Spider graph demonstrating the changes in different aspects of participant confidence to provide different aspects of pain management in primary care.

Overall, levels of knowledge were reasonably good pre-intervention. A significant increase following the intervention was only identified in one domain: supporting patient self-care (increase from 3.84 to 4.19). By contrast, levels of confidence in skills to support self-management were considerably lower at baseline and showed significant increases following the intervention overall (2.87 to 3.60) and across four of the five domains. The only domain where no overall significant improvement was observed was managing patients’ mental health and mood issues but, even here, there was a significant increase in one of the items: sleep issues (3.21 to 3.43). This may have been due to clinicians already having training and a good level of confidence in managing mental health conditions more generally. Some of the largest increases were found in clinicians’ confidence in supporting patients’ self-care (2.11 to 2.87) and managing difficult consultations (from 2.64 to 3.31): see Figures 2 and 3 for a breakdown of knowledge domains and confidence items, respectively.

We noted some differences in confidence between prescribers and non-prescribers. Overall, prescribers felt more confident than non-prescribers to manage many aspects of care. Non-prescribers are less confident to manage issues such as anxiety and stress, depression, anger, and sleep issues but are modestly more confident than prescribers to help manage healthy eating and fitness. Unsurprisingly, non-prescribers felt little confidence in setting up medication reduction plans and were also less confident than prescribers in using pacing activities and preparing setback plans with patients. Prescribers were less confident than non-prescribers when working with angry or demotivated patients.

### Prescription data

**Figure 4.**
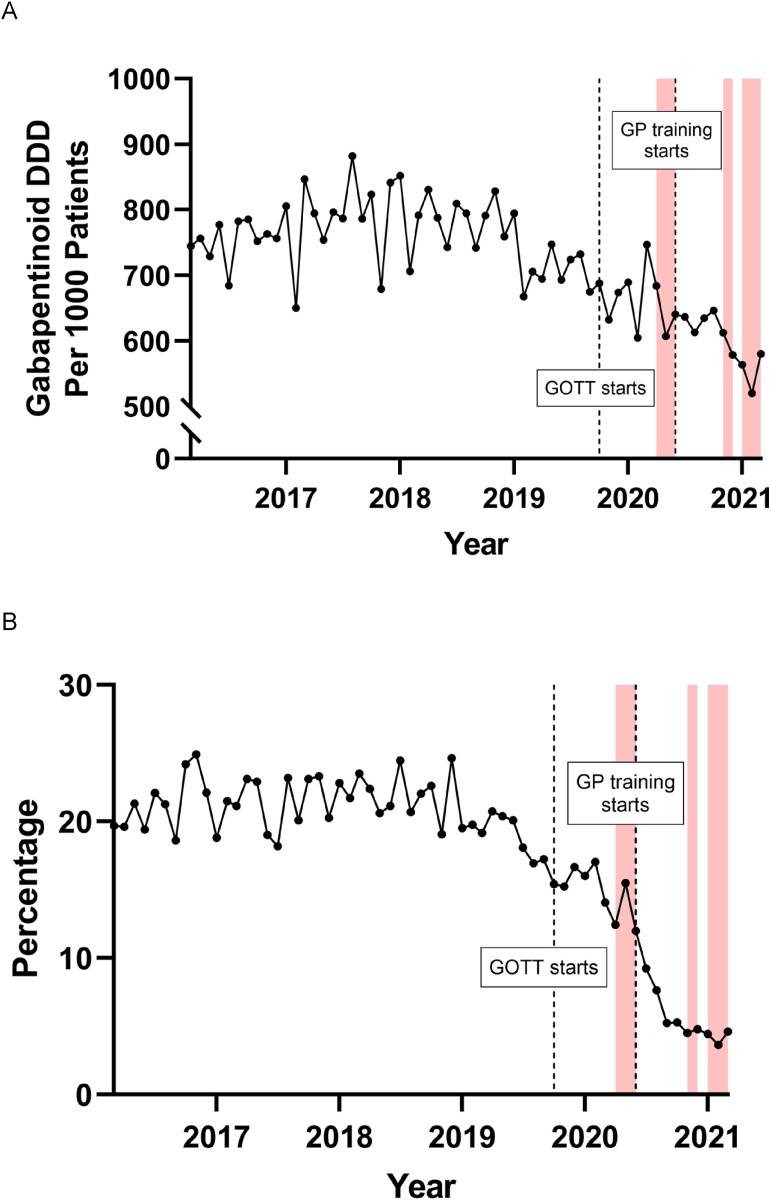
Effects of the GOTT programme at 12 months upon (A) gabapentinoid; (b) opioid prescription rates, gabapentinoid defined daily dose (DDD); percentage high dose: total dose opioid prescriptions. Shaded areas are the three COVID-19 pandemic lockdown periods.

**Figure 5.**
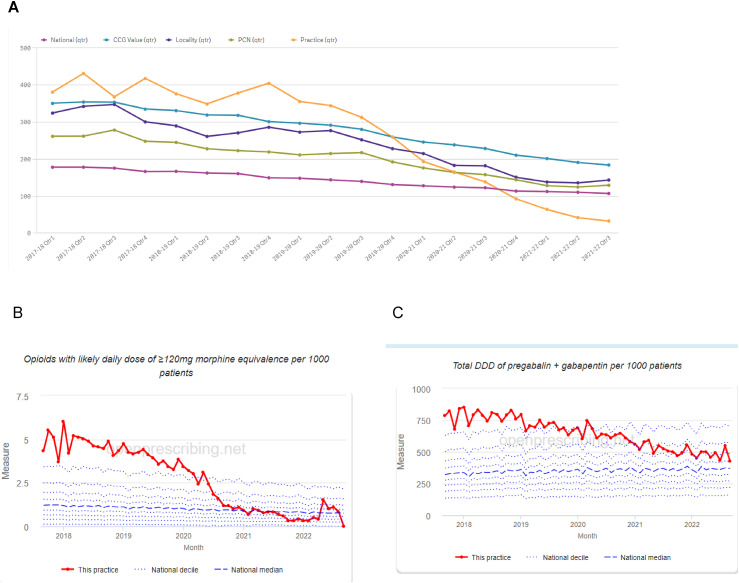
(a) Follow-up performance evaluation of the GOTT programme (orange block) at 30 months: comparison of test case, CCG, and England: (b) Percentage of high dose opioids and (c) defined daily dose (DDD) for gabapentinoids.

Prescription data was recorded after a 12-month study period, with extended follow-up at 30 months (Figures 4 and 5). Note, the GOTT programme was the only intervention available to the practice during this period, mainly due to the pandemic. The GOTT programme facilitated a reduction in high-dose opioids (ca. 90%) and gabapentinoid-defined daily dose (DDD) over the first year of GOTT implementation (ca. 15%) (Figure 4(a) and 4(b)), in particular, following the GOTT 10-Footsteps training programme. The proportion of opioids prescribed as HDOs fell from a mean of 18.36 ± 1.60% before the GOTT programme to 7.37 ± 2.01% in the most recent 3 months. The trends in HDO prescribing were then assessed (Supplement Figure 1(A)). Whilst prescriptions of HDOs were falling before September 2019 with a slope of −0.0719 ± 0.0282, they continued to fall afterwards at a significantly steeper rate than before with a new slope of −0.195 ± 0.0350 and this change in trend was highly significant (ANCOVA, F (1,22) = 7.529, *p* = .0118) (Figure 4, Supplement Figure 2(i)).

Individual changes were investigated to build a more detailed picture of prescription practices (Supplement Figure 2(ii)).

Two notable drug prescription changes occurred during and after the GOTT intervention. Prescriptions of the potent opioid, fentanyl, were rising with a slope of 0.516 ± 0.540 before the GOTT programme but then began to fall with a slope of −1.03 ± 0.418 (Supplement Figure 2) during and after the GOTT intervention, and this change in trend was significant (ANCOVA, F (1,22) = 5.142, *p* = .0335). Gabapentin prescriptions were falling before the programme and after the programme began, but they continued to fall at a significantly faster rate with a change in slope from −1.74 ± 3.36 to −8.01 ± 2.36, during and after the programme (*p* = .03) ((Supplement Figure 2). An attempt was made to assess the changes in individual gabapentinoid prescribing practices, relating to doses. When the gabapentin doses were examined, there was very little change at the highest doses although there was a modest reduction of 2.81% in the proportion of 300 mg prescribed with a similarly sized increase of 3.64% at 100 mg. In contrast, pregabalin showed more changes with a reduction of 9.43% in the proportion of 300 mg prescribed and smaller reductions at 150 mg and 200 mg of 4.90% and 2.77%, respectively. These were associated with increases in the lower doses prescribed with rises of 4.22%, 5.81%, and 6.26% at 50 mg, 75 mg, and 100 mg, respectively. Notably, prescriptions of 300 mg pregabalin, the highest dose, were falling before September 2019 and continued to fall afterwards, but at a significantly faster rate with a highly significant change in slope (ANCOVA, F (1,22) = 15.72, *p* = .0007) from −0.0324 ± 0.0189 to −0.150 ± 0.0228.

Notably, follow-up analysis in December 2022 showed a 100% reduction in high-dose opioids, and a further steady decrease in gabapentinoid DDD (ca.50%). Furthermore, a dramatic reduction of overall opioid prescriptions compared to the local CCG and nationally was observed over the test period (Figure 5(A)).

### Interview analysis

Overall, clinical and non-clinical staff members spoke positively about their experiences of GOTT and were enthusiastic about the potential for the toolkit to help them improve care for patients living with persistent pain. One nurse observed that, *‘it has been good and it has been useful… patients have been positive about it as well’*.

For clinicians it was widely noted that chronic pain consultations can be complex and emotionally demanding, especially in primary care contexts. Patients were sometimes described as being ‘*resistant*’ to advice and difficult to motivate. A couple of clinicians used the term ‘*heartsink patients*’ when describing their own emotional reactions to such cases. In this context, GOTT was seen to provide a useful ‘*structure’* to help both clinicians and patients manage these situations more effectively.

Interviews also corroborated some of the questionnaire findings about gaps in knowledge and confidence around supporting patients living with chronic pain and safe use of medicines, especially in view of the development of the new NICE guidelines,^14^ which the toolkit went some way to addressing. These concerns extended to non-clinical staff, for example, one administrative staff member, referring to processing repeat opioid prescriptions, said, *‘but there’s so many different types of medications, 99% of which I can’t pronounce!’*

Many interviewees expressed concerns about the *practical* implications of implementing the toolkit, particularly when they were already working under considerable time pressures. Several worried that GOTT would add further to demands on their time and make workloads increasingly unmanageable, especially in practices like theirs which supported large numbers of patients with chronic pain. A couple of general practitioners also talked about the increased expectations for them to become ‘*experts*’ in what they regarded as more specialist areas of medicine, beyond their remit as a general practitioner.

The COVID-19 pandemic affected both the implementation of the Toolkit and many aspects of routine patient care. So much of the Toolkit training was delivered online, which suited some staff members (who appreciated the flexibility), but not others, who would have appreciated the opportunity of in-person discussions and shadowing more experienced clinicians. The shift to telephone patient consultations was raised by many interviewees, with some noting that it made pain consultations more difficult, while a couple of GPs had found it easier to have ‘difficult conversations’ (e.g. about drug tapering) by phone rather than face-to-face. When interviewed, some non-clinical reception staff noted that remote appointments can be more accessible for some pain patients who may struggle with mobility. However, they recognised that not all patients had access to the appropriate technology to facilitate this, so some members of these groups might find it much easier to attend face-to-face consultations. Overall, opinions of the effects of the COVID-19 pandemic were mixed, with all staff members being aware of both the positive and negative impacts of more remote consultations and training.

## Discussion

### Key findings

In implementing the GOTT in one large challenging GP practice in a deprived area of Northeast England, notably, with the highest opioid prescription rate in County Durham, we set out to address three main research questions, that we revisit here.

#### Was the implementation of GOTT associated with changes to clinician knowledge and confidence in chronic pain management?

According to the questionnaire data, over the period of the intervention, clinician confidence in chronic pain management increased overall (*p* = .044) and in three of the five domains measured: supporting behaviour change (*p* = .028), supporting self-care (*p* = .008), and managing difficult consultations (*p* = .011). Confidence scores on 8 of the 13 items and the overall confidence in the practice’s ability to work as a team increased significantly between June 2020 and June 2021 (+7.45 points, *p* = .014). This is consistent with a recent social prescribers study using the GOTT 10-Footsteps training programme, where confidence was significantly improved^10^ (Figure 3).

However, while a few of the ‘knowledge’ measures showed improvement over the implementation period, the majority (15/21) showed no significant change, and overall ‘knowledge’ post-intervention was not significantly different from that pre-intervention. It should be noted, however, that pre-intervention knowledge scores were already high, thus leaving less room for improvement in this area and indicating that knowledge alone is not sufficient to implement and to enable person-centred pain self-management techniques (Figure 2).

#### Did opioid and gabapentinoid prescribing practices change over the implementation period?

Pain medicine prescribing behaviours changed substantially over the GOTT test period.

In summary, the main outcome observed was a profound reduction in high-dose opioids (90%), with the largest significant reduction occurring after the training sessions. Gabapentinoid prescription doses also decreased by 15% in one of the highest prescribing practices in the UK, indicating an overall change in how the practice manages chronic pain and prescribing. Importantly, this achievement has been maintained (Figures 4 and 5). Currently, since December 2022, the practice has zero high-dose opioid prescriptions, alongside reduced gabapentinoid prescription doses (ca.50%) (Figure 5(b) and (c)).

All the resources created and advice were available from the GOTT team to the Practice and throughout the UK after the intervention on the https://www.livewellwithpain.co.uk. In fact, top-up training programmes are now available nationwide, through the popular LWWP 10-Footsteps training programme.

#### How was GOTT experienced by all practice staff, and what can we learn from those experiences?

Overall, staff members at the clinic spoke positively about the concept of the GOTT and were enthusiastic about possibilities offered by the toolkit to improve care and safety for patients living with persistent pain. The GOTT helped to offer a clearer structure and process for managing chronic pain and helped clinicians manage consultations more effectively, developing better relationships with patients and leading to better health outcomes. However, some GPs expressed concerns about their capacity to deliver this additional support, noting already high workloads and time constraints as factors allowing partial rather than full engagement with the program. In reality, the GOTT’s aim is to reduce workload by streamlining chronic pain consultations and building better doctor–patient relationships, so this worry is important to note. Two GPs also noted an increasing expectation more generally for GPs to become experts in specialist areas of medicine, despite the fact that pain including chronic pain is one of the commonest reasons for consultations worldwide.^15^

Furthermore, some other (non-GP) staff members felt unsure or were unclear about the purpose and aims of the GOTT as it pertained to their specific role in the practice. Tailored resources for different staff groups with different roles (e.g. administrative staff, nurses, and doctors) were suggested as a potential solution to this. For example, several nurses spoke of experiencing ‘by the way’ consultations whereby patients would come to clinic for one reason (e.g. a smear and a diabetic foot check) and then speak to a nurse about their pain during that consultation. We noted that advice and self-care resources available to use during these ‘by the way’ consultations can help to increase clinician confidence and patient satisfaction in pain management across the practice.

### Strengths and weaknesses of study

To our knowledge, this was the first UK-based study to implement and seek to assess the impacts of a health-focused person-centred pain management intervention of this kind. The combination of multiple data sources (prescription data, measures of clinician knowledge and confidence, and qualitative interview data) is particularly powerful in helping us to understand some of the mechanisms underpinning the observed reduction in opioid and gabapentinoid prescribing in this challenging practice.

The major weakness is that, as a proof-of-concept feasibility study undertaken in a single GP practice with a small sample size and no controls, it is not possible to make definitive causal inferences about the impact of the intervention on changes in prescription rates or measures of knowledge and confidence; neither can we draw wider inferences from the findings to other clinical settings. It should be noted, however, that this was the only intervention utilised by the practice over, and since the test period, the data suggests that scaling up to a trial could be beneficial.

A further significant limitation is the fact that this study coincided with the COVID-19 pandemic – something we could not have anticipated in advance. This had major impacts both on routine care and on the toolkit implementation, which make it difficult to disentangle fully the impacts of GOTT from those of COVID-19. At the time, patients did not have any social prescribing or community resources available, which put further pressure on the GP practice to fill this gap. This further supports the idea that our intervention was the sole reason for the increased GP confidence and dramatic reduction in high-dose opioid prescriptions. Increased work pressures of staff meant some were unable to attend all the training sessions. This also led to a poor response rate to the mid-trial questionnaires.

### Implications and next steps

This study demonstrates that the implementation of a systematic non-pharmacological chronic pain management program is feasible in a primary care setting. Notwithstanding the limitations noted above, there are indications that the deployment of a toolkit like GOTT could have a positive impact on the management of chronic pain in primary care settings, with significantly increased confidence imparted in this challenging GP practice, which had previously the highest opioid prescription rate in County Durham, and a clear effect on the high-dose opioid prescription patient population, which remains at zero to this day. Implementation of the new NICE guidelines makes it particularly important to promote person-centred, non-pharmacological approaches to chronic pain management, and support staff knowledge and skills in primary care settings to deliver these effectively.

Subsequent to our study, a new expanded iteration of the GOTT 10 Footsteps training programme, a co-production of trainers including Lived Experience Trainers (LETs), has gained accreditation by the Personalised Care Institute (PCI) for 2022–2022 and 2023–2024 and has been deployed in other UK regions, including Somerset, Devon, Cornwall, Derby, Birmingham, Durham, London (St Georges Hospital), Berkshire, South Wales, York, and North Tyneside. Evaluations of these programs are currently in progress. Many more regions have approached the team to assist in the implementation of the GOTT 10-Footsteps programme in their CCGs and PCNs. GPs, pharmacists, physiotherapists, and social prescribers are now receiving the training (Corline et al., 2022). The research team also secured an AHSN Bright Ideas in Health Award (2021) evidencing the local impact of, and engagement with, the GOTT. Furthermore, the LWWP 10-Footsteps programme has been promoted widely across the UK [9.10] and is recommended in the recent updated NICE documents for pain management.

The next step is to continue to refine and formally pilot the GOTT intervention and, if successful, to move to a full multi-centre clinical trial, with appropriate control groups. Digitalisation of the programme has been achieved and is currently being trialled in the Derby NHS Foundation Trust. An evaluation is in progress.^16–20^

## Conclusion

This study has provided a mixed-methods assessment of the systematic implementation of a new valuable person-centred non-pharmacological chronic pain management programme (GOTT) in a GP practice in a deprived area of Northeast England. Over the period of implementation, prescription rates of strong opioids and gabapentinoids dropped, and clinicians’ confidence in their skills to support patients with chronic pain in their self-management appeared to increase (although not necessarily their levels of knowledge, which were already good). Staff across the practice (both clinical and non-clinical) spoke positively about the intervention and its potential for improving patient care, albeit with some reservations around workloads and the role of general practice in chronic pain care. Long-lasting positive effects on pain medication reduction have continued subsequent to the implementation year. While we cannot draw definitive causal inferences from this proof-of-concept study, there are strong indications that the deployment of a toolkit could have a positive impact on the person-centred management of chronic pain in primary care settings. Depending on the results of formal pilot and clinical trials, we propose that this could be a game-changer in the non-pharmacological management of chronic pain in the UK.

## Supplemental Material

**Supplemental Material -** Assessing the feasibility of the GOTT (Gabapentinoid and Opioid Tapering Toolkit) in a primary care setting in North-East England Supplemental Material for Assessing the feasibility of the GOTT (Gabapentinoid and Opioid Tapering Toolkit) in a primary care setting in North-East England by Lucy Johnson, Frances Cole, Rebecca Kinchin, Andrea Francis, Konrad Winiarek, Kate Hampshire, and Paul Chazot in British Journal of Pain.
