# Supplementary material for: Assessing the feasibility of the GOTT (Gabapentinoid and Opioid Tapering Toolkit) in a primary care setting in North-East England
Source: Br J Pain. 2024 Oct 20;19(1):29–42. doi: 10.1177/20494637241291534 (PMC11559511; doi:10.1177/20494637241291534)
Supplement: Supplemental Material - Assessing the feasibility of the GOTT (Gabapentinoid and Opioid Tapering Toolkit) in a primary care setting in North-East England [file sj-pdf-1-bjp-10.1177_20494637241291534.pdf]

Supplementary Figure 1
Spearman Rank Regression analysis of the individual pain medicines pre- and post-GOTT programme

i. **High dose opioid prescriptions**

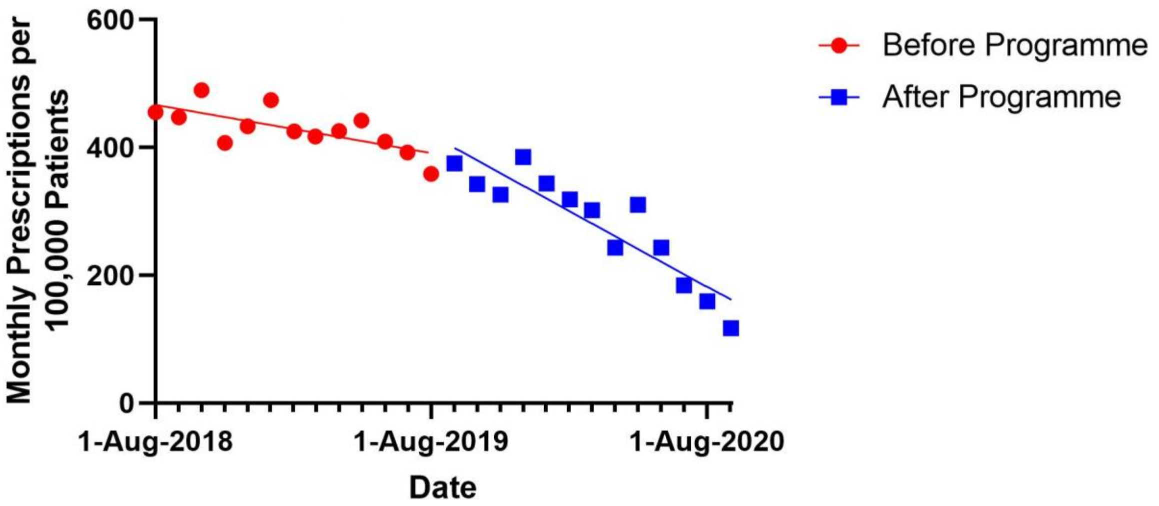

ii. **Prescription rates for two selected individual medications**

a) **Gabapentin**

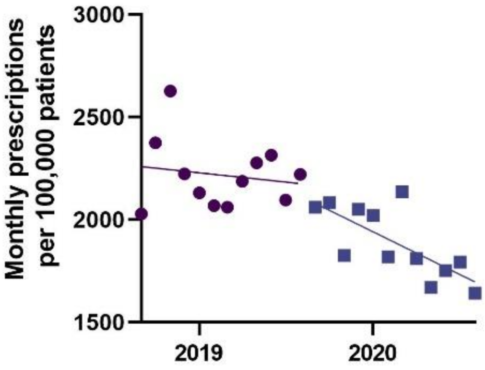

b) **Fentanyl**

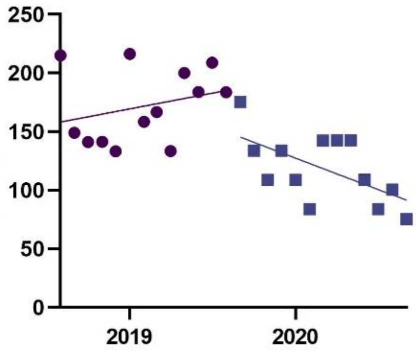

Legend
Trends in the monthly prescriptions per 100,000 patients of two selected medications studied before and after implementation of the GOTT programme.

*(i)**Spearman Rank Regression analysis of the high dose opioid prescription rates**. Whilst prescriptions of HDOs were falling before September 2019, they continued to fall aftewards at a steeper rate than before with a new slope and this change in trend was highly significant (p=0.0118).*
*(ii) **Spearman Rank Regression analysis** (i) **Gabapentin** prescriptions were falling before the GOTT programme and after the programme began, but they continued to fall at a significantly faster rate with a change in slope (p=0.03). (ii) Notably, prescriptions of **Fentanyl** were rising before the GOTT programme, but then began to fall significantly during and after the intervention (0.0335).*

Supplement Figure 2
System One Template used in the GOTT programme development and implementation.

A. **Intervention** used in this study practice known as GOTT (Gabapentinoid and Opioid Toolkit):

- All clinicians received an educational skills programme to support patient pain self-management. This was tailored on the clinicians self-assessment of their learning needs.
- Embedding both clinician skill learning and patient self-care resources for rapid access within consultations into GP clinical management computer system. Relevant pain related clinical resources for self-care support, medicine management shared decision process based on trusted sources including www.livewellwithpain.co.uk were used.
- Such skills and resources were to provide a flexible structural approach to person centred biopsychosocial assessment with reviews of personal health needs and priorities and medicines use due to impact of chronic pain on their health function and so affect safe prescribing behaviour change.
- The primary care computer system, SystmOne template content was created to guide clinicians pain management assessments/reviews (Supplementary Figure 2). It contained
    a. specific clinical record content on ICD-11 pain coding and READ coding, to enable audit of patient group and track change in b. measures.
    b. content to monitor of health function and needs, self-efficacy, medication use and patient goals.
    c. enabled direct links to patient self-care resources with in 'Live Well With Pain' website www.livewellwithpain.co.uk/ and relevant web-based resources including local health and other services. So designed to facilitate rapid clinicians access within the consultation to share with patient.

- Engagement of non-clinical staff to increase knowledge and ways to support patient access to pain management services within the practice.
- Practice prescribing policy for pain medicines was developed and shared with the team clinical and non-clinical team.

GOTT Programme diagram

Assessment for GOTT intervention

Learning needs identified through knowledge and skills self-assessment + qualitative interviews

Identify opioid and gabapentinoid prescribing levels

Self-management and medication management resource needs identified with pracitioners and systematic access through System One GP computer system for practitioner for own learning and for use in consultation (c)

→

GOTT intervention 1

Knowledge and skills training programme over 12 week period consisted of

a. Three online webinars in health coaching in self-management

b. Email to each practitioner

Learning content of two consecutive footsteps of the Ten Footsteps programme at two week intervals with focus on resource and tools.

c. Details of access Live Well with Pain self-management and medicine management resources located in System GP computing system for

1. learning

2. consultation access

3. template for pain management reviews

→

GOTT intervention 2

Developed sppecific System One Computer template for Live Well with Pain Health Check tool create for GOTT intervention that include

Health needs assessment of impac of pain and patient priorities for change

WHO Mental Well-Being

Pain Self-Efficacy 2

Pain Distress /Intensity measures

Medicine management decision tool outcome

Patient goals for self-management

a. Understanding chronic pain

b. Sleep

c. Physical Activity

d. Mood management

e. Plan for medicines

f. Other
